# Intraspecific variation among clones of a naïve rare grass affects competition with a nonnative, invasive forb

**DOI:** 10.1002/ece3.919

**Published:** 2013-12-22

**Authors:** David J Gibson, Justin Dewey, Hélène Goossens, Misty M Dodd

**Affiliations:** Department of Plant Biology Center for Ecology, 1125 Lincoln Avenue, Southern Illinois University CarbondaleCarbondale, Illinois, 62901-6509

**Keywords:** *Alliaria petiolata*, *Calamagrostis porteri* ssp. *insperata*, clones, interspecific interactions, intraspecific variation, non-native species

## Abstract

Intraspecific variation can have a major impact on plant community composition yet there is little information available on the extent that such variation by an already established species affects interspecific interactions of an invading species. The current research examined the competitiveness of clones of a globally rare but locally common native grass, *Calamagrostis porteri* ssp. *insperata* to invasion by *Alliaria petiolata*, a non-native invasive species. A greenhouse experiment was conducted twice over consecutive years in which 15 clones from three populations of *Calamagrostis* were paired with rosettes of *Alliaria* in pots containing native forest soil previously uninvaded by *Alliaria*. Both species showed a negative response to the presence of the other species, although *Alliaria* more so than *Calamagrostis*. Moreover, the effect of *Calamagrostis* depended upon population, and, to a lesser extent, the individual clone paired with *Alliaria*. Competitive effects were stronger in the first experiment compared with when the experiment was repeated in the second year. The influence of *Calamagrostis* clones on the outcome of the experiment varied among populations and among clones, but also between years. Clones from one of the three populations were more influential than clones from the other two populations. Only one of 15 clones, both from the same population, was influential in both experiments. This research supports a growing literature indicating that intraspecific variability among clones of a dominant species can affect interspecific interactions and that such variability in a native species can affect performance of an invading species.

## Introduction

Understanding the importance of individual variation on population and community dynamics, and the upscaling of processes at the level of individuals to population scale patterns are key ecological challenges (Sutherland et al. 2013). In this vein, intraspecific variation among competing neighbors is known to affect species diversity in plant communities (Booth and Grime 2003; Vellend 2006; Gibson et al. 2012). These effects arise as intraspecific variability mediates local-scale interspecific interactions (Turkington 1996). A consequence of these interactions is that potential niche space availability may vary depending upon the presence or number of certain genotypes of dominant species in the community. These dominant species are those that make a substantial contribution to plant biomass in a community by virtue of their large relative size and high frequency of occurrence. The mass ratio hypothesis (MRH) states that the traits and functional diversity of dominant species in a community play a primary role in development of community and ecosystem function (Grime 1998). Thus, if a dominant species in the community exhibits ecologically relevant intraspecific variability, then the opportunity for establishment of a new species in a community may depend upon the particular genotypes of the dominant species that are present (Crutsinger et al. 2010; Adams et al. 2011).

It has been suggested that genetic diversity and the occurrence of species modulates invasion resistance (Crutsinger et al. 2008; Vellend et al. 2010) and can be considered a development of Elton's diversity-resistance hypothesis (Elton 1958). Infact, recent plant community models place primacy on the importance of genetic variability affecting the competitive ability of dominant species as determinants of invasion resistance (Vellend 2006; Gibson et al. 2012). While these models consider the outcome of these interactions on native species diversity (Fridley et al. 2007; Whitlock et al. 2007, 2011; Fridley and Grime 2010), less is known about how intraspecific variability of a dominant species affects competitive response and effect (sensu Goldberg and Landa 1991) with invasive species. The expectation from these conceptual models and the well-established principles of natural selection (Darwin 1859) is that some individuals in a native species population would be better competitors against an invasive non-native species than others.

In this study, we challenged individuals of the exotic, non-native (to North America) invasive species *Alliaria petiolata* (Bieb.) Cavara and Grande (hereafter *Alliaria*) with clones of a globally rare, perennial grass, *Calamagrostis porteri* ssp. *insperata* (Swallen) C. Greene (hereafter *Calamagrostis*) collected from three separate populations in Illinois, USA. We tested the hypothesis that the competitive effect and response (sensu Goldberg and Landa 1991) of *Calamagrostis* on *Alliaria* would be affected by differences among *Calamagrostis* populations or clones within each population, or both. *Alliaria* is highly competitive in mixtures against some native North American species (Meekins and McCarthy 1999), but has yet to invade *Calamagrostis* populations. *Calamagrostis* has not been exposed to *Alliaria* and is thus a naïve competitor with respect to *Alliaria* thereby removing the potential bias of competition between an exotic and a native species after the opportunity for either to have been subject to selection imposed by one on the other (Strauss et al. 2006). *Calamagrostis* populations have a low genetic diversity (Esselman et al. 1999) making the potential importance of significant effects of individual clones on competitive outcome between these two species more relevant than if we had ‘stacked the deck’ by carrying out this experiment with a highly diverse native species. When the genetic diversity of one of an interacting species pair is low, then genotype effects should be correspondingly low in comparison with interactions among species with high genetic diversity.

## Materials and Methods

### Study species

*Calamagrostis porteri* ssp. *insperata* (Poaceae) is a cool-season, loosely clumped, perennial grass limited in its global distribution to approximately 80 populations in five US states where it is considered endangered in Arkansas, Illinois, Kentucky, Maryland, Ohio, and Tennessee, threatened in Indiana. *Calamagrostis* occurs in forest openings and upland oak woodland throughout its range. Where *Calamagrostis* populations occur, the plant occurs as common scattered clumps in the forest understory. Vegetative growth is generally vigorous and determined by light, humidity, and soil temperature, with the rare flowering events requiring high early-mid growing season light and soil moisture (Bittner and Gibson 1998; Gibson et al. 2009). When *Calamagrostis* plants do flower, less than 1% of florets produce viable seed (Havens and Holland 1998). Molecular analysis of the Illinois populations of *Calamagrostis* based on ISSR markers showed high within-population genetic similarity, and low among-population genetic similarity (Marriage 2002). Despite low within-population genetic diversity, *Calamagrostis* clones 1 m or more apart are genetically unique likely reflecting relictual diversity from more frequent sexual reproduction in the past or more recent somatic mutation.

*Alliaria petiolata* (Brassicaceae) is a highly competitive, self-compatible, invasive non-native biennial herb widespread in deciduous forest understories and forest edges throughout the eastern North America. Native to Europe, molecular studies indicate that *Alliaria* appears to have been introduced multiple times to the US in the early 19th century (Durka et al. 2005), and it was first observed in southern Illinois in 1988 (Gibson et al. 2006), but has yet to invade any of the three *Calamagrostis* populations in the state despite being recorded from one of the sites (i.e., Lusk Creek Canyon Natural Area). Germinating in early spring, first year plants overwinter as a basal rosette before bolting, flowering and setting seed in late-spring before dying. *Alliaria* competes against native plants through early season flowering and seed set ahead of most native plants (Anderson et al. 1996), and the production of allelochemicals which can disrupt mycorrhizal associations (Stinson et al. 2006; Wolfe et al. 2008) and suppress native species recruitment (McCarthy 1997; Prati and Bossdorf 2004; Stinson et al. 2007).

### Greenhouse experiment

Fifteen to seventeen *Calamagrostis* clones from each of the three Illinois populations at Bell Smith Springs Ecological Area (BSS: 37°31'16”N, 88°39'42”W), Lusk Creek Canyon Natural Area (LC: 37°30'57”N, 88°32'26”W), and Hays Creek Canyon (HC: 37°29'12”N, 88°36' 6”W) were haphazardly selected and collected in mid-summer 2002. Each clone that was collected consisted of a clump of physically connected rhizomes, roots, and attached vegetative tillers. Clumps were separated by at least 5 m and prior genetic profiling of the populations (Marriage 2002) and subsequent reaction norms on greenhouse grown clones (Gibson et al. 2009) indicated that the clumps were most likely separate genotypes. Thereafter, the clones were maintained and subdivided as necessary in individual 17.7-cm by 15.2-cm pots in a greenhouse to obtain genetically identical clonal replicates. The plants were periodically clipped to 9 cm above the soil surface and repotted so that individuals did not become root bound in the pots. Samples of these clones were used in a greenhouse and field experiment to test the effects of light availability and soil moisture (Gibson et al. 2009). There were no observable negative effects on the clones maintained under these conditions from the time of collection (2002) until the experiment was established (2008–2009). In October 2008, shortly before establishment of this experiment, the *Calamagrostis* plants were treated for leaf spot with the systemic benzimidazole fungicide Benomyl; the presence of pathogens was not observed thereafter.

Vegetative first year rosettes of *Alliaria* were collected from a large population (several hundred individuals) on the Southern Illinois University Carbondale campus, Jackson County, Illinois in early autumn 2008 and 2009. These plants were placed within 24 hr into experimental pots.

**Figure 1:**
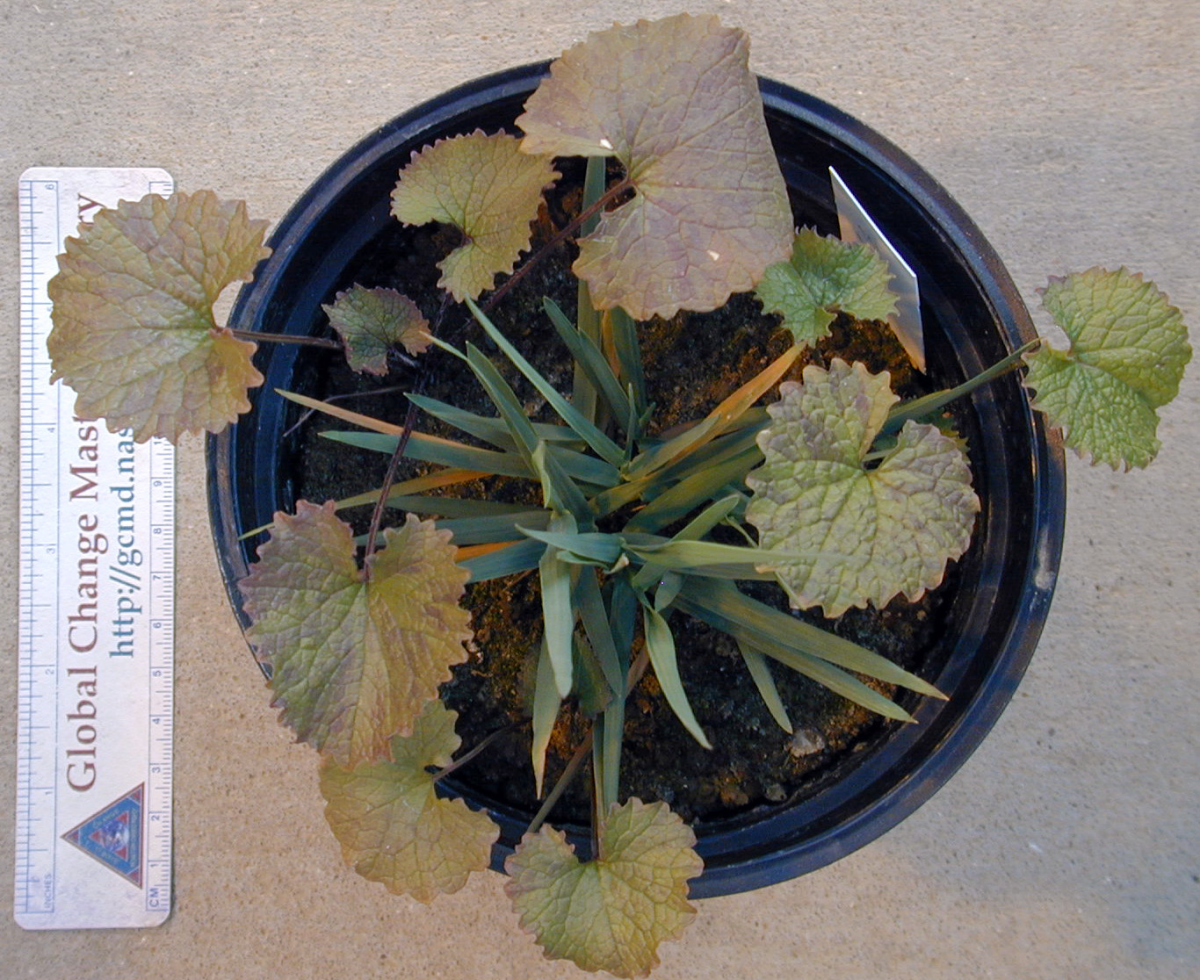
An experimental unit showing a single clone of *Calamagrostis porteri* ssp. *insperata* growing in the center of a 15-cm-diameter pot surrounded by three plants of *Alliaria petiolata*.

The experiment was established in 15-cm-diameter plastic pots filled with a 50:50 mixture of forest soil and sterilized masonry sand. The soil:sand mixture had pH 5.3, CEC 13.2 meq/100 g, 1.2% organic matter, 90 μg L^−1^ P, 132 μg L^−1^ K, 1493 μg L^−1^ Ca, and 376 μg L^−1^ Mg (A&L Analytical Laboratories, Inc., Memphis, Tn). The forest soil was collected from a *Quercus velutina/Q. alba* dominated area on the SIUC campus >100 m from the nearest *Alliaria* population. A pairwise competition experimental design was established (Gibson 2002) with monocultures of single *Calamagrostis* clones (7–23 tillers each when planted) or three *Alliaria* rosettes (1–8 leaves per plant) randomly assigned to each pot and mixtures of a central *Calamagrostis* clone surrounded by three *Alliaria* rosettes (Fig. 1). This design has proven to be useful for investigating invasive-native species interactions (Vilà and Weiner 2004) as well as pairwise competitive interactions in a variety of controlled settings (Gibson et al. 1999; Connolly et al. 2001). Experimental treatments were *Calamagrostis* source population (*n* = 3), *Calamagrostis* clone (*n* = 5 per source population), and competition (monoculture of *Calamagrostis* or *Alliaria*, or mixture). The particular *Calamagrostis* clones chosen for this experiment from the collection maintained in the greenhouse were randomly chosen from those with sufficient replicates of approximately the same size. There were 3 replicates per treatment combination giving a total of 135 experimental units randomly located by replicate on one of three greenhouse benches. Pots were bottom watered daily with an automated watering system with supplemental top watering by hand as necessary to main adequate soil moisture and avoid drought stress. The experiment was conducted twice to investigate consistency of clonal effects on competition with *Alliaria*. *Calamagrostis* plants were given a 2-week establishment period in the pots before *Alliaria* plants were planted. In year 1, *Calamagrostis* was planted November 10 to November 11, 2008 with *Alliaria* planted into pots on November 17, 2008. This experiment ran through May 29, 2009 when a final harvest was conducted. In year 2, *Calamagrostis* was planted September 29 through October 2, 2009, with *Alliaria* planted on October 13, 2009. Final harvest for the year 2 experiment was April 22 to April 30, 2010. Eight *Alliaria* plants died by February 16 in year 1 and these were not replaced. However, mortality was higher in year 2 and 77 dead plants were replaced through January 6, 2010. Occasional outbreaks of aphids and white flies on the *Alliaria* were controlled by spraying with the insect growth regulator Enstar® (active ingredient (S)-kinoprene) and the pyrethroid insecticide Talstar® (active ingredient bifenthrin). The experiment was conducted in the SIUC Tree Improvement Center Greenhouse. Temperature and supplemental greenhouse lighting were controlled to mimic late autumn sunlight and temperature conditions, which ranged 13–30 °C over the course of the experiments.

Several response variables were measured to capture the dynamics of competitive interactions (Gibson et al. 1999; Trinder et al. 2013): On *Calamagrostis* clones, tiller counts and leaf number per plant were recorded at approximately three weekly intervals five times over the course of the experiment each year. For *Alliaria*, leaf number, leaf width (maximum width of largest leaf), plant height per plant, and the number of surviving plants per pot (of three planted) were recorded five times. At final harvest, individual plants were washed free of soil, separated into above-and belowground tissues, dried at 55 °C for >48 h, and weighed.

### Data analysis

Growth of *Calamagrostis* and *Alliaria* was investigated separately on the mean value of response variables per pot with a mixed model analysis using proc MIXED in SAS, version 9.2 (SAS Institute Inc. 2002-2008) testing the main effects and the interaction of *Calamagrostis* source population (*n* = 3) and competition treatment (*n* = 2), with the main effect of *Calamagrostis* clone nested within source population. A repeated measures mixed model including the effect of time of measurement (days or weeks since start of the experiment) was conducted on variables measured over time. Model degrees of freedom were estimated using the Satterthwaite approximation, or the Kenward-Roger correction for repeated measures, and the appropriate covariance structure were incorporated in the analysis following Littell et al. (2006). The three greenhouse benches that pots were placed on were included as random factors. To run a full mixed model, it was necessary to assign pots without *Calamagrostis*, that is., *Alliaria* monoculture controls to a *Calamagrostis* source population and clone. This assignment was made randomly ten separate times with the mixed model being run following each assignment. Average F statistics for each treatment combination obtained from each of the ten random assignments and model runs was retained and tested for significance at α = 0.05. In addition, a restricted analysis of the effect of *Calamagrostis* clone (nested in source population) on mixtures where *Alliaria* was paired in a pot with a specific *Calamagrostis* clone (i.e., without monocultures) was undertaken to more precisely investigate the clone effect. Results of both the full model and the restricted model are reported. Separate analyses were undertaken for the experiments in each year (hereafter year 1 and year 2).

Cook's D influence statistics were calculated using the influence option of proc MIXED in SAS, version 9.2 (SAS Institute Inc. 2002-2008) to determine the *Calamagrostis* clone that was most influential on plant performance (i.e., a response variable) that the mixed models identified as showing a significant *Calamagrostis* clone effect on either *Calamagrostis* or *Alliaria*. Cook's D statistic (Cook 1977) is used in the detection of influential observations in linear regression and linear mixed models (e.g., Schowalter et al. 1991) and is calculated as the distance between original log likelihoods based on all observations and on removing, in this case, one *Calamagrostis* clone at a time from the dataset. D statistics of *Calamagrostis* clones exceeding the 90th percentile of all calculated values were considered to indicate that a clone had a significant effect on the analysis based upon the particular response variable being tested.

## Results

### Competitive response of *Calamagrostis* to *Alliaria*

Regardless of whether plants were competing with *Alliaria* or not, *Calamagrostis* clones and populations grew significantly different to each other in both years of the experiment (Table 1). Recognition of interclonal or interpopulation differences depended upon the growth parameter and was not consistent from the first to the second year (Figs 3). For example, BSS clones did not differ in terms of aboveground biomass in year 1, but showed clear differences among clones in year 2. Similarly, no populations showed differences among clones in terms of belowground biomass in year 1, whereas clones from all three populations exhibited differences in year 2. Clone H2 from HC was generally the largest clone regardless of response variable measured over the two years. There was not a clearly identifiable low performing clone. Overall, plants from BSS were larger than plants from HC, which were generally larger than plants from LC (Figs 3). However, in year 2, HC plants had more tillers (21.0 ± 1.1) and leaves (66.3 ± 3.7) per plant than plants from the other populations (tillers: BSS 16.2 ± 1.1, LC 15.5 ± 1.0; leaves: BSS 46.8 ± 2.7, LC 46.8 ± 2.67).

**Table 1:** Competitive response of *Calamagrostis* to *Alliaria*. F statistics from mixed model analysis testing the effects of *Calamagrostis* source population (P), clone (nested within population), and competition (C) with or without *Alliaria* through time (T) on final biomass and leaf and tiller number of *Calamagrostis*.

		Biomass			
	NumDF/DenDF	Aboveground	Belowground	Leaf number	Tiller number
Year 1					
Population (P)	2/70.7, 52.1	7.92***	3.84	3.46*	3.32*
Competition (C)	1/70.7, 52.1	13.73***	1.39	0.88	0.18
P ^*^ C	2/70.7, 52.1	1.35	1.99	0.65	0.60
Time (T)	4/333, 333			919.89***	556.47***
P ^*^ T	8/333, 333			0.87	1.09
C ^*^ T	4/333, 333			1.45	3.60**
P ^*^ C ^*^ T	8/333, 333			0.75	0.99
Clone	12/70.7, 52.1	1.92*	1.35	1.85†	1.21
Year 2					
Population (P)	2/59.6, 62.2	8.84***	0.19	7.18**	7.31**
Competition (C)	1/59.2, 59.0	2.92†	0.58	0.52	0.07
P ^*^ C	2/59.1, 58.9	0.56	2.22	0.04	0.51
Time (T)	3/221, 229			128.65***	205.72***
P ^*^ T	6/221, 229			3.50**	1.62
C ^*^ T	3/221, 229			0.94	0.83
P ^*^ C ^*^ T	6/221, 229			1.60	1.44
Clone	12/58.8, 59.7	3.25***	3.97***	2.31*	2.04*

Num DF, numerator degrees of freedom. Denominator degrees of freedom (Den DF) = 65 for analysis without repeated measures, and as shown following NumDF for leaf number and tiller number analyzed with repeated measures analysis, respectively.

† *P* < 0.1.

* *P* < 0.05.

** *P* < 0.01.

*** *P* < 0.001.

**Figure 2:**
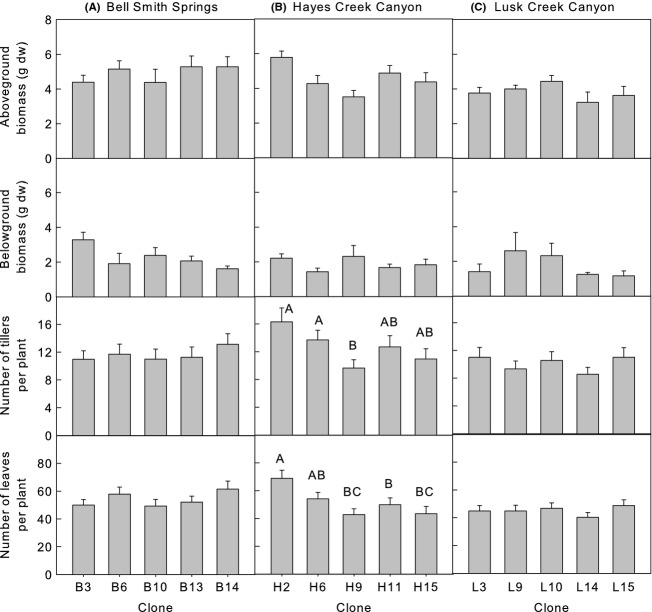
Mean (± SE) *Calamagrostis* response by population and clone, year 1, (A) Bell Smith Springs, (B) Hayes Creek Canyon, and (C) Lusk Creek Canyon. Bars sharing the same letter, or no letters, among clones and within populations are not significantly different (*P *=* *0.05).

**Figure 3:**
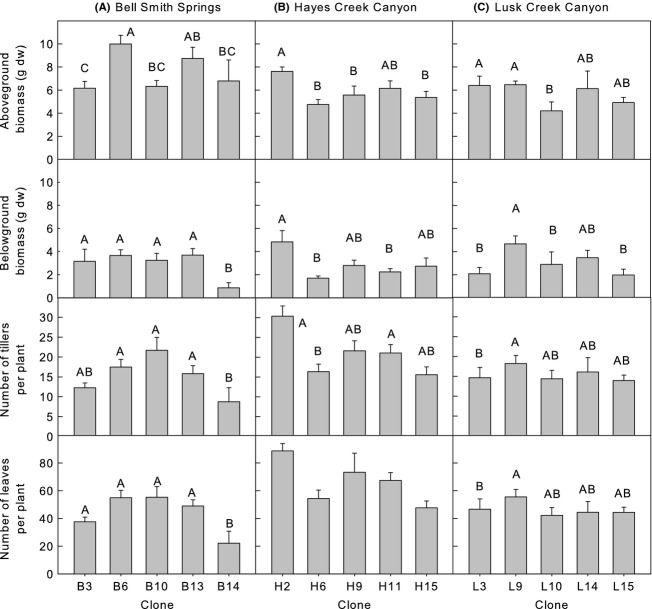
Mean (± SE) *Calamagrostis* response by population and clone, year 2, (A) Bell Smith Springs, (B) Hayes Creek Canyon, and (C) Lusk Creek Canyon. Bars sharing the same letter, or no letters, among clones and within populations are not significantly different (*P *=* *0.05).

A response of *Calamagrostis* to competition with *Alliaria* occurred in both years affecting aboveground biomass and number of tillers per plant in year 1 and marginally affecting aboveground biomass in year 2 (Table 1). In all cases, where a response was observed, the presence of *Alliaria* reduced growth of *Calamagrostis* (Figs 5), although the response illustrated by tiller number was only evident at the end of the experiment in year 1 (Fig. 5).

**Figure 4:**
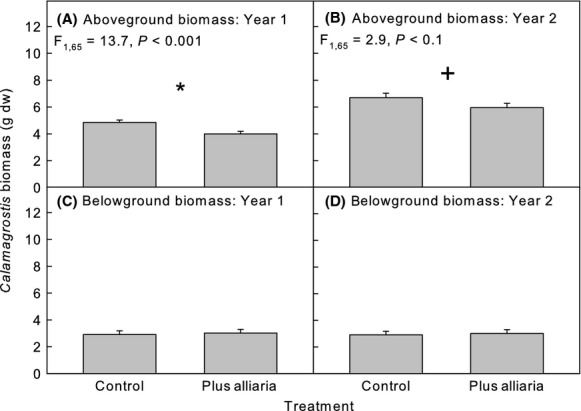
Mean (± SE) *Calamagrostis* response to competition treatments (Control = *Calamagrostis* monoculture, Plus Alliaria = *Calamagrostis-Alliaria* mixture; (A, B) aboveground biomass, and (C, D) belowground biomass in years 1 and 2, respectively. Symbols above pairs of bars indicate a significant difference between the means (^+^*P *<* *0.1, *P <* *0.05); on bars with no symbols there were no differences among treatments.

**Figure 5:**
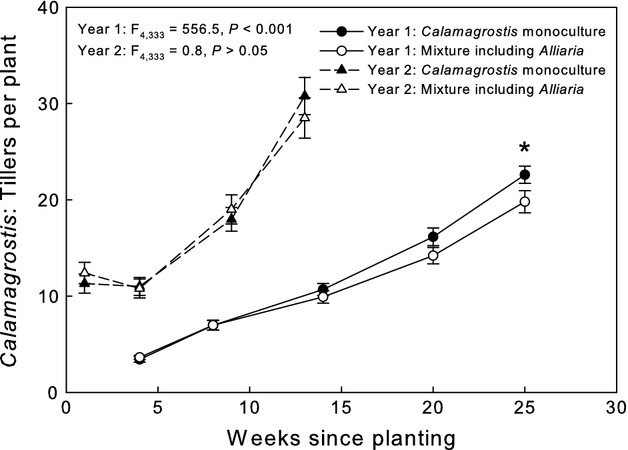
Mean (± SE) *Calamagrostis* tiller number in response to competition treatments (*Calamagrostis* monoculture and in mixture including *Alliaria*) through time. There was a significant difference in tiller number per *Calamagrostis* plant in monoculture versus in mixture with *Alliaria* 25 days after planting in year (*P <* *0.05).

### Competitive effect of *Calamagrostis* on *Alliaria*

**Figure 6:**
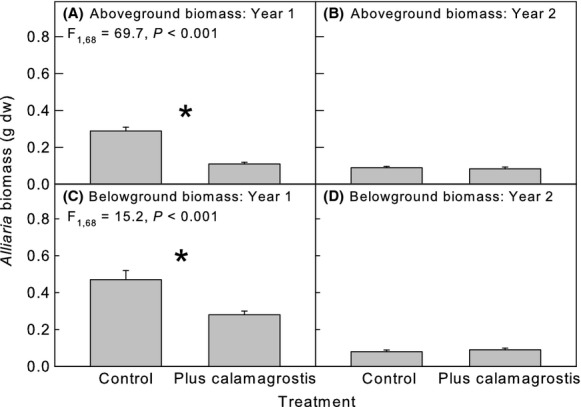
Effect of *Calamagrostis* competition (Control = *Alliaria* monoculture, Plus *Calamagrostis* = mixture including *Calamagrostis*) on *Alliaria* mean (± SE) (A, B) aboveground biomass, and (C, D) belowground biomass in years 1 (A, B) and 2 (B, D). Symbols above pairs of bars indicate a significant difference between the means (*P <* *0.05); on bars with no symbols there were no differences among treatments.

**Figure 7:**
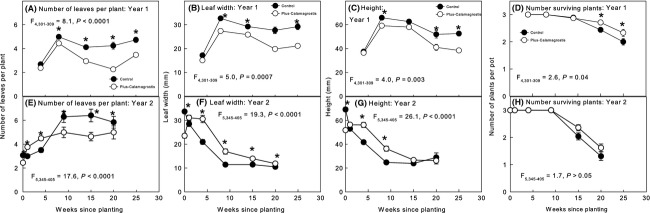
Effect of *Calamagrostis* competition (Control = *Alliaria* monoculture, Plus *Calamagrostis* = mixture including *Calamagrostis*) on *Alliaria* [mean (± SE)] in years 1 and 2, (A, E) number of leaves per plant; (B, F) leaf width; (C, G) height, and (D, H) number of surviving *Alliaria* plants per pot. A * symbol above pairs of points indicate a significant difference in mean values at the number of weeks since planting shown (*P <* *0.05).

The full mixed model suggested that neither *Calamagrostis* population nor clones within populations affected *Alliaria* growth in either year of the experiment (Table 2). However, the restricted model analysis showed that *Calamagrostis* clone nested within population affected *Alliaria* leaf width and height in year 1 and number of leaves per plant in year 2 (Table 2, and see Appendices S1 and S2). Competition with *Calamagrostis* regardless of population or clone reduced growth of *Alliaria* in year 1 affecting all measured variables (Fig. 6). In year 2, *Alliaria* plants grown in competition with *Calamagrostis* had fewer, larger leaves, and showed higher survivorship (Fig. 7) compared with *Alliaria* plants grown in the absence of *Calamagrostis*. Final biomass of *Alliaria* was unaffected by *Calamagrostis* (Fig. 6). Overall, *Alliaria* plants in year 2 were smaller than plants in year 1. Biomass of *Alliaria* in year 1 was twice that of *Alliaria* in year 2 and was reduced by competition in year 1 but not year 2.

**Table 2:** Competitive effect of *Calamagrostis* on *Alliaria*. F statistics from repeated measures mixed model analysis testing the effects of *Calamagrostis* population source (P), clone (nested within population), and competition (C) on leaf number, leaf width, height, and number of living plants through time (T), and final above-and belowground biomass of *Alliaria*. The restricted *Calamagrostis* clone analysis tested the subset of the experimental pots in which both *Alliaria* and *Calamagrostis* were planted together (i.e., not including the *Calamagrostis* or *Alliaria* monocultures).

		Biomass					
	Num DF/Den DF	Aboveground	Belowground	Leaf number	Leaf width	Height	Number of living *Alliaria* plants
Year 1							
Population (P)	2/69–124	0.72	0.79	0.20	1.21	1.37	1.03
Competition (C)	1/69–124	69.66***	15.23***	55.89***	62.66***	29.30***	2.42
P ^*^ C	2/69–124	0.84	1.53	0.19	1.61	1.24	1.12
Time (T)	4/301–309			67.84***	98.93***	85.37***	43.75***
P ^*^ T	8/69–124			0.46	0.78	0.59	0.92
C ^*^ T	4/301–309			8.11***	4.95***	3.99**	2.55*
P ^*^ C ^*^ T	8/312–334			0.62	0.81	0.71	1.12
*Calamagrostis* clone	12/72–128	0.71	0.62	0.46	1.16	0.99	0.73
*Calamagrostis* clone restricted analysis	12/30.9–55.9	2.14*	0.66	0.76	2.20*	2.10*	1.15
Year 2							
Population (P)	2/73–189	0.82	1.99	0.65	0.25	0.61	0.36
Competition (C)	1/72–189	0.42	0.59	2.01	10.36**	1.92	1.00
P ^*^ C	2/72–189	1.06	2.06	1.08	0.34	0.63	0.31
Time (T)	5/361–395			29.06***	86.00***	89.51***	103.38***
P ^*^ T	10/385–405			1.42	1.55	1.23	1.05
C ^*^ T	5/343–396			17.59***	19.26***	26.06***	1.66
P ^*^ C ^*^ T	10/370–405			1.26	1.57	1.35	0.85
*Calamagrostis* clone	12/71–198	1.20	1.20	1.22	0.88	0.74	0.88
*Calamagrostis* clone restricted analysis	12/30.6–54.9	1.46	0.94	1.93*	0.91	0.56	0.46

Num DF, numerator degrees of freedom. Denominator degrees of freedom (Den DF) = 68 for analysis without repeated measures, and the range of degrees of freedom values estimated using the Kenward-Roger correction for leaf number, leaf width, height, and number of living plants analyzed with repeated measures analysis are shown following Num DF.

* *P* < 0.05.

** *P* < 0.01.

*** *P* < 0.001.

### Competitive Influence of *Calamagrostis* clones

Cook's D statistics identified the most influential *Calamagrostis* clones (highest Cook's D statistics) either increasing or decreasing performance in the experiment more so than other clones. *Calamagrostis* clones from BSS were more influential in affecting growth of *Calamagrostis* in intraspecific mixture or *Alliaria* in interspecific mixtures with *Calamagrostis* than were clones from HC or LC (Table 3). Five of the fifteen *Calamagrostis* clones were identified as being influential (with Cook's D statistics exceeding the 90th percentile of values; Table 3). Four of these clones were from BSS (i.e., clones B6, B10, B13, and B14), one from HC (clone H6), and none from LC. However, of these, only clones B6, B10, and B14 from BSS affected more than one growth parameter. *Alliaria* was affected by three BSS clones (B10, B13, and B14), one HC clone (H6), but no LC clones. The *Calamagrostis* clones identified as having a significant influence did not necessarily reduce growth. For example, the *Calamagrostis* clone with the largest influence on belowground biomass of *Calamagrostis* (clone B14) had the lowest biomass in mixture compared with other clones from the BSS population (Fig. 3). There was little evidence of year-to-year consistency among the *Calamagrostis* clones identified as being the most influential in affecting growth with only one of fifteen clones (B10) being influential in affecting performance of *Calamagrostis* in both years. No *Calamagrostis* clones were influential in affecting growth of *Alliaria* in both years.

**Table 3:** Cook's D statistics indicating influence of *Calamagrostis* clones on *Calamagrostis* and *Alliaria* response variables that affected significantly performance (*Calamagrostis* clone effect in Tables 1 and 2). Cook's D statistics exceeding the 90th percentile (i.e., D ≥ 0.20) of all values are in bold.

	*Calamagrostis*						*Alliaria*			
	Year 1		Year 2				Year 1			Year 2
Clone	Aboveground biomass	Leaf number	Aboveground biomass	Belowground biomass	Leaf number	Tiller number	Aboveground biomass	Leaf width	Height	Leaf number
Bell Smith Springs										
B3	0.03	0.08	0.17	0.08	0.07	0.07	0.01	0.03	0.03	0.06
B6	0.06	0.14	**0.20**	0.12	**0.40**	**0.27**	0.05	0.02	0.04	0.05
B10	**0.23**	0.12	0.11	0.01	**0.22**	**0.28**	0.14	**0.26**	**0.29**	0.13
B13	0.01	0.05	0.03	0.04	0.05	0.11	**0.54**	0.06	0.06	0.10
B14	0.07	0.03	0.01	**0.26**	0.18	0.08	<0.01	0.03	0.03	**0.28**
Hayes Creek										
H2	0.15	0.13	0.16	0.07	0.03	0.04	0.15	0.02	0.01	0.09
H6	0.12	0.05	0.04	0.05	0.06	0.04	0.15	0.05	0.01	**0.24**
H9	0.11	0.09	0.08	<0.01	0.14	0.02	0.03	<0.01	<0.01	0.16
H11	0.01	0.05	0.01	0.04	0.02	0.03	0.01	0.02	0.01	0.07
H15	0.13	0.14	0.01	<0.01	0.03	0.07	0.07	0.03	0.01	0.16
Lusk Creek										
L3	0.04	0.07	0.01	0.08	0.13	0.16	0.01	0.06	0.08	0.06
L9	0.04	0.11	0.09	0.15	0.05	0.04	0.04	0.08	0.06	0.03
L10	0.05	0.07	0.09	0.09	0.12	0.11	0.03	0.05	0.05	0.05
L14	0.18	0.16	0.12	0.02	0.05	0.08	0.33	0.10	0.10	0.04
L15	0.17	0.09	0.03	0.08	0.05	0.10	0.49	0.18	0.19	0.03

### Year-to-year ranking in growth

**Figure 8:**
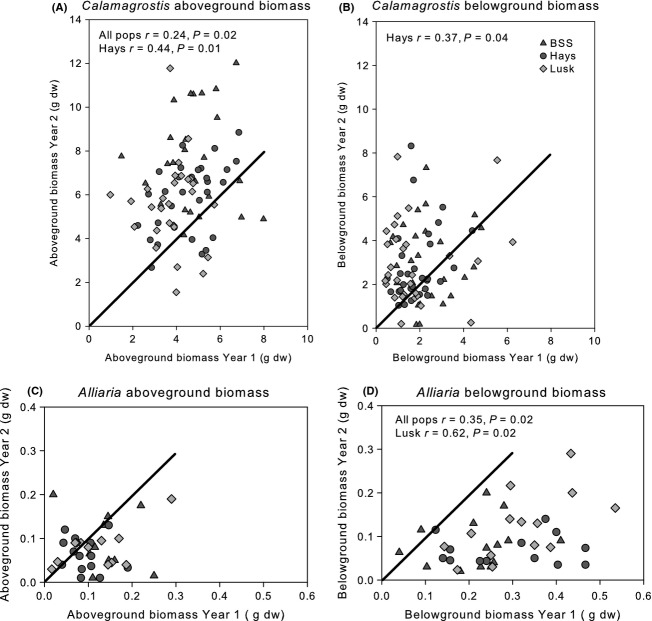
Relationship between aboveground (A, C) biomass and belowground (B, D) biomass of (A, B) *Calamagrostis* and (C, D) *Alliaria* between year 1 and year 2 by source population of *Calamagrostis* (BSS = Bell Smith Springs, Hays = Hays Creek Canyon, Lusk = Lusk Creek Canyon). The solid line shows the 1:1 biomass line between year 1 and year 2. Plants on the line had the same biomass in year 1 and year 2. r and *P*-values shown for significant correlations only (*P *<* *0.05) for all populations of *Calamagrostis* considered together (“all pops”) or by source population.

Regardless of competitive environment, there was a positive correlation between aboveground biomass of *Calamagrostis* clones from year 1 to year 2 across all populations (Spearman's paired rank correlation *r *=* *0.24, *P *=* *0.02, Fig. 8A), and for clones from HS in terms of both aboveground and belowground biomass (*r *=* *0.44, 0.37, respectively, *P *<* *0.05, Fig. 8A,B). In other words, the largest *Calamagrostis* clones in year 1 were also the largest clones in year 2, and *vice versa*. Similarly, belowground, *Alliaria* grew best in the presence of the same set of *Calamgrostis* clones in both years. Competitive effect of *Calamagrostis* clones on belowground, but not aboveground biomass of *Alliaria* was positively correlated from year 1 to year 2 (*r *=* *0.35, *P *=* *0.02, Fig. 8C,D) across all populations. Within *Calamagrostis* populations, the effect of *Calamagrostis* clones on belowground biomass of *Alliaria* was restricted to clones from LC (*r *=* *0.62, *P *=* *0.02, Fig. 8D).

## Discussion

Intraspecific variability is increasingly recognized as an important internal biotic filter structuring communities (Violle et al. 2011). As part of an emerging community genetics paradigm (Hersch-Green et al. 2011), intraspecific variability adds to niche complexity and can allow coexistence of more species in a community than under strict models based upon concepts of limiting similarity (Abrams 1983; Gibson et al. 2012). In terms of invasion biology, intraspecific variability in members of the native plant community can show differential response to and effects on individual invader species, including *Alliaria* as we show here. Indeed, evolution of resistance by native species to invaders occurs at the population level (Rowe and Leger 2011), including for *Alliaria* (Cipollini and Hurley 2008). Ultimately, intraspecific variability in native species can affect resistance to invasion both in terms of the individual invader species and the resulting community (Crutsinger et al. 2008).

Intraspecific variability can affect neighboring species through variation in ecologically relevant morphological or physiological traits that contribute to competitive interactions (Johnson et al. 2008; Kotowska et al. 2010; Whitlock et al. 2010). In the current study, using a random subset of the clones used in Gibson et al. (2009), we show significant differences among clones of *Calamagrostis* regardless of whether they were competing with *Alliaria* or not. The ranking of growth of *Calamagrostis* clones was consistent in both years of the experiment (Fig. 8) as was the effect of *Calamagrostis* clonal identity on belowground biomass of *Alliaria*. When *Alliaria* was present in mixture, growth of *Calamagrostis* was reduced consistent with *Alliaria*'s known role as a highly competitive invasive species (Meekins and McCarthy 1999). In addition, *Calamagrostis* generally reduced *Alliaria* growth in mixture, although this effect was not consistent from 1 year to the next and could not be consistently related to a particular *Calamagrostis* clone. A stronger effect of *Calamagrostis* was among source populations where clones from the Bell Smith Springs population had a larger effect on *Alliaria* than clones from the other two populations. These results suggest that while there is phenotypic intraspecific variation in *Calamagrostis*, its effect on potentially invading *Alliaria* may be greater among than within populations. Thus, we would predict that of the Illinois populations of *Calamagrostis*, the Bell Smith Springs population would show greater resistance to invasion by *Alliaria* than the Lusk Creek or Hayes Creek Canyon populations. This result is consistent with the proposition that operational importance of intraspecific variance is scale related (Violle et al. 2011).

Repeating the experiment a second year was informative because it showed that *Calamagrostis* clones that were most influential in affecting competitive response and effect were not consistent from 1 year to the next. This inconsistency and differential response among the clones could be due to uncontrolled differences in the greenhouse conditions from 1 year to the next, or differences among the genotypes of *Alliaria* used in the experiment from 1 year to the next. Certainly, invasive species, including *Alliaria*, are reported to evolve rapidly as they invade new habitat (Müller-Schärer et al. 2004). Although *Alliaria* plants were taken from the same source population each year, as a biennial plant we would have been collecting from a new and different generation of plants in the second year. However, our source *Alliaria* population would not have been subject to selection from *Calamagrostis* as the nearest population was > 69 km away, but they could nevertheless, have evolved in response to other selection pressures (Cipollini 2002; Bossdorf et al. 2005). Such a response of *Alliaria* over a 2-year time period may seem unlikely but would reflect rapid evolution in populations of this invading species. Regardless, the implication is that competitive response of an invasive plant to variability in native plants in the habitat being invaded may be contingent upon response to current environmental conditions (Goldberg 1996).

Mechanistically, we can speculate why some clones of *Calamagrostis* had more influence than others on the outcome of competition against *Alliaria*. Both above-and belowground biomass of *Calamagrostis* was reduced in the presence of *Alliaria*. However, *Alliaria* grows as a basal rosette that, except when the flower stem bolts, only minimally overtops and shades the tussock of upright leafy tillers of *Calamagrostis* (Fig. 1). In year 2, there was reduced aboveground biomass of *Alliaria* associated with the two *Calamagrostis* clones that had the largest aboveground biomass (Figs 7, and see Appendix S1). Interspecific competition with *Alliaria* may occur principally belowground as it can compete against neighbors through allelopathic disruption of the mycorrhizal network (Cantor et al. 2011; Hale et al. 2011) through the action of various flavonoids and glycosides (Cipollini et al. 2008). The susceptibility of *Calamagrostis porteri* to allelopathically mediated competitive effects is unknown but could vary among clones. The congener *C. canadensis* is susceptible to allelopathic compounds (Winder 1997). Thus, some *Calamagrostis* clones may be better able to respond to competition against *Alliaria* than other clones within the context of allelopathic interactions. An alternative mechanism might simply be that the largest clones of *Calamagrostis* demand the largest amount of soil moisture in a pot effectively drawing down moisture below the level required by *Alliaria* for it to compete effectively (Meekins and McCarthy 2000). The converse mechanism is that intraspecific allopathic variation in the invader, *Alliaria* in this case, differentially affects invasive species success (Lankau 2011).

Regardless of the possible mechanisms of competitive interactions involved in the experiment presented here, we show that in a species of low genetic variability (*Calamagrostis*), there is nonetheless sufficient intraspecific genotypic variation to elicit differences in competitive effect and response. Even though the genetic variation effects were limited and varied between 1 year of the experiment and the next, the occurrence of variation both within and among populations suggests that invasion success of an exotic (*Alliaria* here) at the plant-to-plant neighborhood scale is likely to vary depending upon the competitiveness of individual genotypes that are encountered, and when they are encountered. As *Alliaria* continues to spread and if it reaches *Calamagrostis* populations, invasion success may be determined by success against particular genotypes of *Calamagrostis* through alteration of plant–soil feedbacks (Suding et al. 2013). Moreover, the genetic variability in competitiveness among individuals of *Calamagrostis* that we demonstrate may also be more affective in interactions with co-occurring native species than we found against the exotic *Alliaria*. In addition, the importance of intraspecific variation in competitiveness may be more relevant in widespread, more genetically diverse species than in the rare *Calamagrostis* studied here. Regardless, as noted by Wiens (1977), competition may well be temporarily sporadic, but as shown here, genotypically dependent as well.

## Data Accessibility

Plant data for *Calamagrostis* and *Alliaria* for both years of the experiment have all been deposited in Dryad (http://datadryad.org) (DOI: 10.5061/dryad.k68n1).

## Supporting Information

**Appendix S1.** *Calamagrostis* effect by population and clone on Alliaria: Year 1. Bars above clones within a population sharing the same letter were not significantly different (P < 0.05). **Appendix S2.** *Calamagrostis* effect by population and clone on Alliaria: Year 2. Bars above clones within a population sharing the same letter were not significantly different (P < 0.05).

## References

[b1] Abrams P (1983). The theory of limiting similarity. Annu. Rev. Ecol. Syst.

[b2] Adams RI, Goldberry S, Whitham TG, Zinkgraf MS, Dirzo R (2011). Hybridization among dominant tree species correlates positively with understory plant diversity. Am. J. Bot.

[b3] Anderson RC, Dhillion SS, Kelley TM (1996). Aspects of the ecology of an invasive plant, Garlic Mustard (*Alliaria petiolata*), in Central Illinois. Restor. Ecol.

[b4] Bittner RT, Gibson DJ (1998). Microhabitat relations of the rare reed bent grass, *Calamagrostis porteri* subsp. *insperata* (Poaceae), with implications for its conservation. Ann. Mo. Bot. Gard.

[b5] Booth RE, Grime JP (2003). Effects of genetic impoverishment on plant community diversity. J. Ecol.

[b6] Bossdorf O, Auge H, Lafuma L, Rogers WE, Siemann E, Prati D (2005). Phenotypic and genetic differentiation between native and introduced plant populations. Oecologia.

[b7] Cantor A, Hale A, Aaron J, Traw MB, Kalisz S (2011). Low allelochemical concentrations detected in garlic mustard-invaded forest soils inhibit fungal growth and AMF spore germination. Biol. Invasions.

[b8] Cipollini D (2002). Variation in the expression of chemical defenses in *Alliaria petiolata* (Brassicaceae) in the field and common garden. Am. J. Bot.

[b9] Cipollini KA, Hurley SL (2008). Variation in resistance of experienced and naïve seedlings of Jewelweed (*Impatiens capensis*) to invasive garlic mustard (*Alliaria petiolata*. Ohio J. Sci.

[b10] Cipollini D, Stevenson R, Cipollini K (2008). Contrasting effects of allelochemicals from two invasive plants on the performance of a nonmycorrhizal plant. Int. J. Plant Sci.

[b11] Connolly J, Wayne P, Bazzaz FA (2001). Interspecific competition in plants: how well do current methods answer fundamental questions?. Am. Nat.

[b12] Cook RD (1977). Detection of influential observations in linear regression. Technometrics.

[b13] Crutsinger GM, Souza L, Sanders NJ (2008). Intraspecific diversity and dominant genotypes resist plant invasions. Ecol. Lett.

[b14] Crutsinger GM, Strauss SY, Rudgers JA (2010). Genetic variation within a dominant shrub species determines plant species colonization in a coastal dune ecosystem. Ecology.

[b15] Darwin C (1859). The origin of species by means of natural selection.

[b16] Durka W, Bossdorf O, Prati D, Auge H (2005). Molecular evidence for multiple introductions of garlic mustard (*Alliaria petiolata*, Brassicaceae) to North America. Mol. Ecol.

[b17] Elton CS (1958). The ecology of invasion by plant and animals.

[b18] Esselman EJ, Jianqiang L, Crawford DJ, J. L W, A. D W (1999). Clonal diversity in the rare *Calamagrostis porteri* spp. *insperata* (Poaceae): comparative results for allozymes and random amplified polymorphic DNA (RAPD) and intersimple sequence repeat (ISSR) markers. Mol. Ecol.

[b19] Fridley JD, Grime JP (2010). Community and ecosystem effects of intraspecific genetic diversity in grassland microcosms of varying species diversity. Ecology.

[b20] Fridley JD, Grime JP, Bilton M (2007). Genetic identity of interspecific neighbours mediates plant responses to competition and environmental variation in a species-rich grassland. J. Ecol.

[b21] Gibson DJ (2002). Methods in comparative plant population ecology.

[b22] Gibson DJ, Connolly J, Hartnett DC, Weidenhammer JD (1999). Designs for greenhouse studies of interactions between plants. J. Ecol.

[b23] Gibson DJ, Battaglia LL, Inczauskis JR, Hacker M (2006). http://opensiuc.lib.siu.edu/pb_reports/1.

[b24] Gibson DJ, Delong M, Chandy S, Honu YAK (2009). Reproductive challenges of a rare grass, *Calamagrostis porteri* subsp. *insperata* (Swallen) C. Greene: implications for habitat restoration. Appl. Veg. Sci.

[b25] Gibson DJ, Allstadt A, Baer SG, Geisler M (2012). Incorporating the genetic diversity of foundation species into community assembly and diversity. Oikos.

[b26] Goldberg DE (1996). Competitive ability: definitions, contingency and correlated traits. Phil. Trans. Roy. Soc. B-Biol. Sci.

[b27] Goldberg DE, Landa K (1991). Competitive effect and response: hierarchies and correlated traits in the early stages of competition. J. Ecol.

[b28] Grime JP (1998). Benefits of plant diversity to ecosystems: immediate, filter and founder effects. J. Ecol.

[b29] Hale AN, Tonsor SJ, Kalisz S (2011). Testing the mutualism disruption hypothesis: physiological mechanisms for invasion of intact perennial plant communities. Ecosphere.

[b30] Havens K, Holland DL (1998). Factors affecting reproductive success in a rare grass, *Calamagrostis porteri* subsp. *insperata*. Ann. Mo. Bot. Gard.

[b31] Hersch-Green EI, Turley NE, Johnson MTJ (2011). Community genetics: what have we accomplished and where should we be going?. Philoso. Transac. Royal Soci. B-Biol. Sci.

[b32] Johnson MTJ, Dinnage R, Zhou AY, Hunter MD (2008). Environmental variation has stronger effects than plant genotype on competition among plant species. J. Ecol.

[b33] Kotowska AM, Keddie JF, Cahill Jr BA (2010). Plant genetic diversity yields increased plant productivity and herbivore performance. J. Ecol.

[b34] Lankau RA (2011). Intraspecific variation in allelochemistry determines an invasive species' impact on soil microbial communities. Oecologia.

[b35] Littell RC, Milliken GA, Stroup WW, Wolfinger RD, Schabenberger O (2006). SAS® for mixed models.

[b36] Marriage T (2002).

[b37] McCarthy BC, Luken JO, Thieret JW (1997). Response of a forest understory community to experimental removal of an invasive nonindigenous plant (*Alliaria petiolata*, Brassicaceae). Assessment and management of plant invasions.

[b38] Meekins JF, McCarthy BC (1999). Competitive ability of *Alliaria petiolata* (Garlic Mustard, Brassicaceae), an invasive, nonindigenous forest herb. Int. J. Plant Sci.

[b39] Meekins JF, McCarthy BC (2000). Responses of the biennial forest herb *Alliaria petiolata* to variation in population density, nutrient addition and light availability. J. Ecol.

[b40] Müller-Schärer H, Schaffner U, Steinger T (2004). Evolution in invasive plants: implications for biological control. Trends Ecol. Evol.

[b41] Prati D, Bossdorf O (2004). Allelopathic inhibition of germination by *Alliaria petiolata* (Brassicaceae). Am. J. Bot.

[b42] Rowe CLJ, Leger EA (2011). Competitive seedlings and inherited traits: a test of rapid evolution of Elymus multisetus (big squirreltail) in response to cheatgrass invasion. Evol. Appl.

[b43] SAS Institute Inc (2002). The SAS System for Windows, Version 9.2.

[b44] Schowalter TD, Sabin TE, Stafford SG, Sexton JM (1991). Phytophage effects on primary production, nutrient turnover, and litter decomposition of young Douglas-fir in western Oregon. For. Ecol. Manage.

[b45] Stinson KA, Campbell SA, Powell JR, Wolfe BE, Callaway RM, Thelen GC (2006). Invasive plant suppresses the growth of native tree seedlings by disrupting belowground mutualisms. PLoS Biol.

[b46] Stinson K, Kaufman S, Durbin L, Lowenstein F (2007). Impacts of Garlic Mustard invasion on a forest understory community. Northeast. Nat.

[b47] Strauss SY, Lau JA, Carroll SP (2006). Evolutionary responses of natives to introduced species: what do introductions tell us about natural communities?. Ecol. Lett.

[b48] Suding KN, Stanley Harpole W, Fukami T, Kulmatiski A, MacDougall AS, Stein C (2013). Consequences of plant–soil feedbacks in invasion. J. Ecol.

[b49] Sutherland WJ, Freckleton RP, Godfray HCJ, Beissinger SR, Benton T, Cameron DD (2013). Identification of 100 fundamental ecological questions. J. Ecol.

[b50] Trinder CJ, Brooker RW, Robinson D (2013). Plant ecology's guilty little secret: understanding the dynamics of plant competition. Funct. Ecol.

[b51] Turkington R (1996). Intergenotypic interactions in plant mixtures. Euphytica.

[b52] Vellend M (2006). The consequences of genetic diversity in competitive communities. Ecology.

[b53] Vellend M, Drummond EBM, Tomimatsu H (2010). Effects of genotype identity and diversity on the invasiveness and invasibility of plant populations. Oecologia.

[b54] Vilà M, Weiner J (2004). Are invasive plant species better competitors than native plant species? – evidence from pair-wise experiments. Oikos.

[b55] Violle C, Enquist BJ, McGill BJ, Jiang L, Albert CH, Hulshof C (2011). The return of the variance: intraspecific variability in community ecology. Trends Ecol. Evol.

[b56] Whitlock R, Grime JP, Booth RE, Burke T (2007). The role of genotypic diversity in determining grassland community structure under constant environmental conditions. J. Ecol.

[b57] Whitlock R, Grime JP, Burke T (2010). Genetic variation in plant morphology contributes to the species-level structure of grassland communities. Ecology.

[b58] Whitlock R, Bilton MC, Grime JP, Burke T (2011). Fine-scale community and genetic structure are tightly linked in species-rich grasslands. Phil. Trans. Roy. Soc. B-Biol. Sci.

[b59] Wiens JA (1977). On competition and variable environments. Am. Sci.

[b60] Winder RS (1997). The in vitro effect of allelopathy and various fungi on marsh reed grass (*Calamagrostis canadensis*. Can. J. Bot.

[b61] Wolfe BE, Rodgers VL, Stinson KA, Pringle A (2008). The invasive plant *Alliaria petiolata* (garlic mustard) inhibits ectomycorrhizal fungi in its introduced range. J. Ecol.

