# Supplementary material for: Intraspecific variation among clones of a naïve rare grass affects competition with a nonnative, invasive forb
Source: Ecol Evol. 2013 Dec 22;4(2):186–99. doi: 10.1002/ece3.919 (PMC3925382; doi:10.1002/ece3.919)

Appendix S1. *Calamagrostis* effect by population and clone on *Alliaria*: Year 1. Bars above clones within a population sharing the same letter were not significantly different (P <0.05).

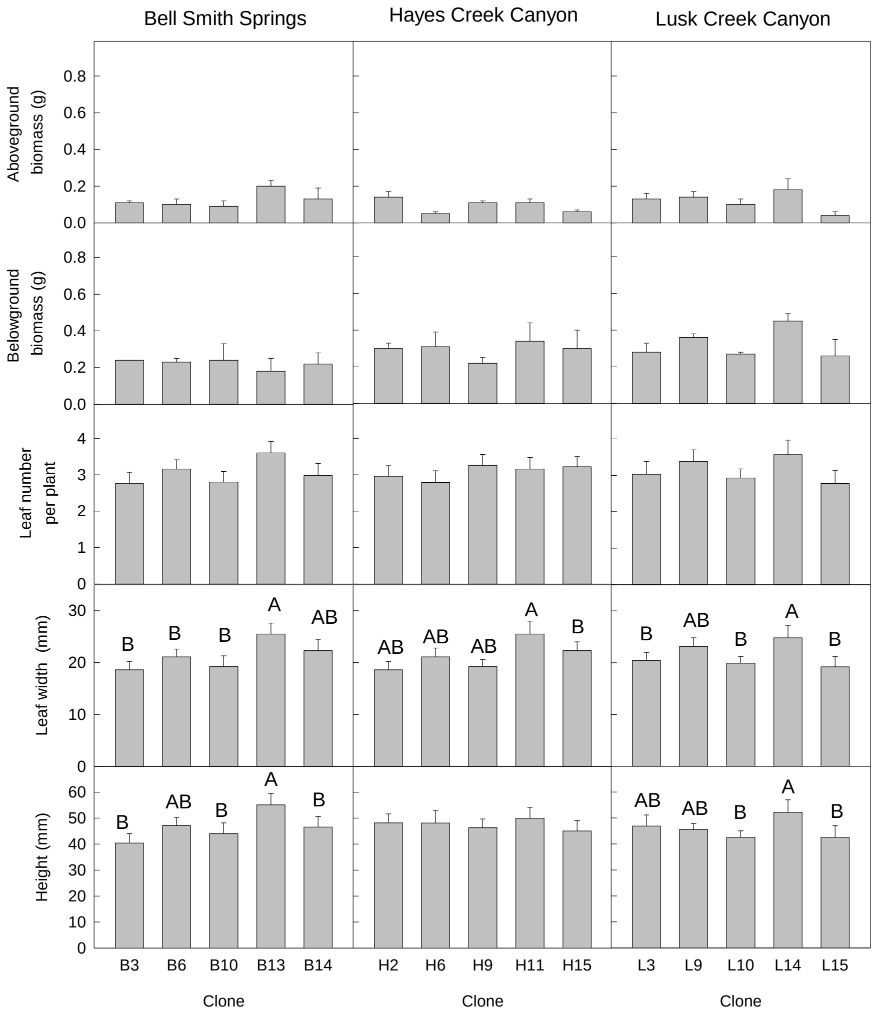

Appendix S2: *Calamagrostis* effect by population and clone on *Alliaria*: Year 2. Bars above clones within a population sharing the same letter were not significantly different (P <0.05).

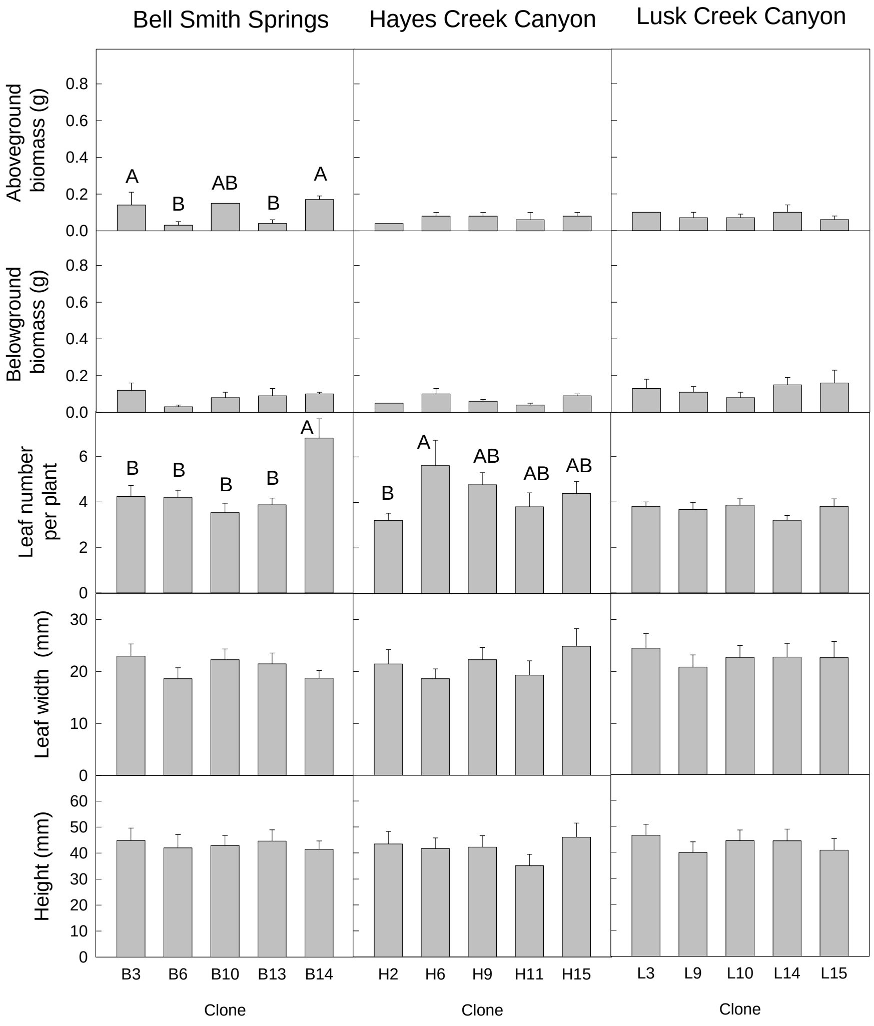

Supplement: Supplementary file 1 — Appendix S1. Calamagrostis effect by population and clone on Alliaria: Year 1. Bars above clones within a population sharing the same letter were not significantly different (P < 0.05). Appendix S2. Calamagrostis effect by population and clone on Alliaria: Year 2. Bars above clones within a population sharing the same letter were not significantly different (P < 0.05). [file ece30004-0186-sd1.pdf]
